# The TRPC1 Ca^2+^-permeable channel inhibits exercise-induced protection against high-fat diet-induced obesity and type II diabetes

**DOI:** 10.1074/jbc.M117.809954

**Published:** 2017-10-26

**Authors:** Danielle Krout, Anne Schaar, Yuyang Sun, Pramod Sukumaran, James N. Roemmich, Brij B. Singh, Kate J. Claycombe-Larson

**Affiliations:** From the ‡ Grand Forks Human Nutrition Research Center, U.S. Department of Agriculture, Agricultural Research Service (USDA-ARS), Grand Forks, North Dakota 58203 and; § Department of Biomedical Sciences, School of Medicine and Health Sciences, University of North Dakota, Grand Forks, North Dakota 58203

**Keywords:** calcium, diabetes, exercise, obesity, transient receptor potential channels (TRP channels), SOCE, TRPC1

## Abstract

The transient receptor potential canonical channel-1 (TRPC1) is a Ca^2+^-permeable channel found in key metabolic organs and tissues, including the hypothalamus, adipose tissue, and skeletal muscle. Loss of TRPC1 may alter the regulation of cellular energy metabolism resulting in insulin resistance thereby leading to diabetes. Exercise reduces insulin resistance, but it is not known whether TRPC1 is involved in exercise-induced insulin sensitivity. The role of TRPC1 in adiposity and obesity-associated metabolic diseases has not yet been determined. Our results show that TRPC1 functions as a major Ca^2+^ entry channel in adipocytes. We have also shown that fat mass and fasting glucose concentrations were lower in TRPC1 KO mice that were fed a high-fat (HF) (45% fat) diet and exercised as compared with WT mice fed a HF diet and exercised. Adipocyte numbers were decreased in both subcutaneous and visceral adipose tissue of TRPC1 KO mice fed a HF diet and exercised. Finally, autophagy markers were decreased and apoptosis markers increased in TRPC1 KO mice fed a HF diet and exercised. Overall, these findings suggest that TRPC1 plays an important role in the regulation of adiposity via autophagy and apoptosis and that TRPC1 inhibits the positive effect of exercise on type II diabetes risk under a HF diet-induced obesity environment.

## Introduction

Intracellular Ca^2+^ signaling has been suggested as playing several important roles in regulating cellular energy metabolism (1); however, the specific Ca^2+^ ion channels involved have not yet been identified. Transient receptor potential canonical channel-1 (TRPC1)^4^ functions as a Ca^2+^ entry channel that is found in key metabolic tissues, including the hypothalamus (2), adipose tissue (3), and skeletal muscle (4), making it a likely candidate for the regulation of cellular energy metabolism. As such, functional disturbance of TRP family channels could play a role in regulating adiposity and obesity-related conditions such as insulin resistance (5–7). However, the exact role of TRPC1 in adipose tissue mass changes, development of obesity, and obesity-associated metabolic disease risks has not yet been determined.

TRP channels contain six hydrophobic stretches and a pore loop motif intercalated between the fifth and sixth transmembrane segments (8, 9). The mammalian TRP channel family consists of subfamilies of classical TRP channels (TRPC1–TRPC7), vanilloid receptor-related TRP channels (TRPV1–TRPV6), melastatin-related TRP channels (TRPM1–TRPM8), and polycystin-related TRP channels (TRPP1–TRPP2) (10). Of these, several TRPC channels are activated by G protein-coupled receptors and receptor tyrosine kinases that are linked to phosphoinositide hydrolysis via phospholipase C activation, whereas other TRPC channels (specifically TRPC1 and TRPC4) are activated upon depletion of intracellular Ca^2+^ stores (9, 10).

Obesity is a hallmark of metabolic syndrome and a key feature of obesity is the disruption of metabolic homeostasis leading to excess adipose accumulation (11–14), thus therapeutic targeting of proteins involved in these pathways could be essential for slowing or preventing the development of obesity and obesity-related health problems, including insulin resistance and type II diabetes. TRPC1 gene expression is induced in differentiated adipocytes (3), yet no data are currently available on whether TRPC1 has a role in adipocyte energy metabolism regulation by altering mitochondrial energy oxidation, adipocyte lipid storage or size, and adipose tissue weight.

One way that TRP channels may control energy metabolism and adiposity is by acting as sensors for chemical factors necessary in adipocyte biology (3). Dietary saturated fat intake promotes obesity and type II diabetes (15) whereas n-3 polyunsaturated fatty acids (PUFAs) mainly found in fish oil produce opposite effects (16, 17). Treatment of human embryonic kidney cells (HEK 293) with n-3 PUFAs such as linolenic, docosahexaenoic, and eicosapentaenoic acids inhibit Ca^2+^ entry via TRPC5 homomeric and TRPC1–TRPC5 heteromeric channels (3). Interestingly, the PUFA concentrations used in this study were within the physiologically achievable range of the human diet (3). Whether high dietary saturated fat intake modulates adipocyte energy metabolism via TRPC1-mediated signaling is not yet known.

Moreover, experimental evidence indicates that several TRP channels play an important role in the onset of diabetes (5–7) or diet-induced obesity (18); however, the role of TRPC1 in these circumstances is not yet established. Exercise regulates body energy stores and insulin resistance by reducing adipocyte size and lipid content (19, 20) and by regulating serum glucose homeostasis through inducing glucose transporter type 4 (GLUT4) protein expression (21). Interestingly, treadmill running prevents Ca^2+^ dysregulation and diabetic dyslipidemia in HF fed swine (22). TRPC1 knock-out (KO) mice with attenuated Ca^2+^ entry (23) experienced reduced muscular endurance due in part to reduced force production and a greater rate of muscle fatigue (4). However, whether a HF diet could exacerbate reduced exercise tolerance in TRPC1 KO mice or contribute to mitochondrial energy metabolism dysfunction is also not yet known. Currently, no other studies have investigated the effects of dietary HF and exercise on adipocyte energy metabolism alteration via TRPC1 protein regulation of intracellular Ca^2+^ homeostasis.

The present study investigated the involvement of TRPC1 in diet-induced obesity and type II diabetes. Additionally, regulation of adipocyte formation under normal-fat (NF) (21) or HF diet and control cage or voluntary exercise conditions was also evaluated to determine how optimal dietary treatments and exercise promote a healthy body weight. Our data indicate that TRPC1 KO mice fed a HF diet and exercised are protected from diet-induced obesity and type II diabetes risk indicative of an underlying mechanism resulting from loss of Ca^2+^ influx through TRPC1 that mediates a reduction in adiposity and insulin resistance when HF diet and exercise are combined.

## Results

### Expression and characterization of calcium channels in subcutaneous adipose tissue

**Figure 1.**
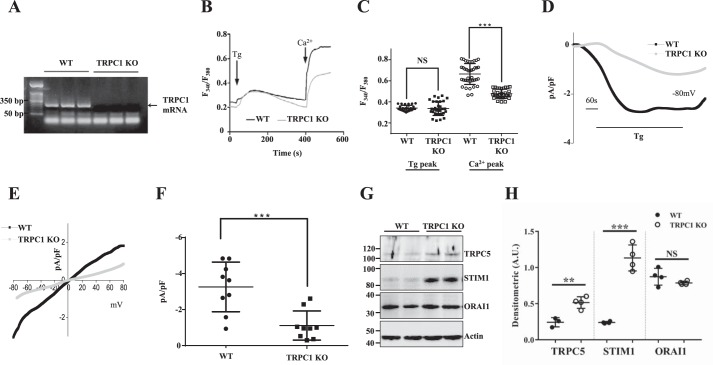
**TRPC1 expression and Ca^2+^ signaling are decreased in subcutaneous adipose tissue of TRPC1 KO mice.** *A*, RT-PCR expression of TRPC1 from subcutaneous adipose tissue of WT and TRPC1 KO mice following 12 weeks of diet and exercise. *B*, analog plots of the fluorescence ratio (340/380) from an average of 30–40 cells in each condition. *C*, quantification (mean ± S.D.) of 340/380 ratio. *** indicates significance (*p* < 0.001) *versus* control. *D*, thapsigargin (*Tg*)-induced currents were evaluated in adipocytes obtained from WT and TRPC1 KO mice. The holding potential for current recordings was −80 mV. *E* and *F*, I/V curves (mean current) under these conditions and the average (8–10 recordings) current intensity under various conditions is shown in *F*. *** indicates values (mean ± S.D.) that are significantly different from control (*p* < 0.001). *G*, subcutaneous adipose tissue from WT and TRPC1 KO mice were resolved and analyzed by Western blotting using antibodies labeled in the figure with β-actin as a loading control. *H*, quantification of each protein. Data are presented as mean ± S.D., *n* = 4. **, *p* < 0.01; ***, *p* < 0.001.

We first examined TRPC1 transcripts in subcutaneous adipose tissue from WT and TRPC1 KO mice. Adipose tissues were obtained from both WT and TRPC1 KO mice and mRNA was isolated, after which RT-PCR confirmed that full-length TRPC1 expression is lost in KO mice (Fig. 1*A*). We next investigated Ca^2+^ entry upon store depletion using primary adipocyte cells. Endoplasmic reticulum (ER) Ca^2+^ stores were depleted by the addition of thapsigargin (Tg) (2 μm), a SERCA (sarcoplasmic/endoplasmic reticulum Ca^2+^-ATPase) pump blocker, which activates store-mediated Ca^2+^ entry. In the absence of extracellular Ca^2+^, the increase in intracellular Ca^2+^ ([Ca^2+^]*_i_*) evoked by thapsigargin (first peak) was unaltered in TRPC1 KO cells when compared with WT control cells (Fig. 1, *B* and *C*). Subsequently, addition of external Ca^2+^ (1 mm), which initiates store-mediated Ca^2+^ entry, was significantly decreased in adipocytes obtained from TRPC1 KO mice (Fig. 1, *B* and *C* and supplemental Fig. 1*A*). Similarly, we also depleted internal stores through angiotensin II, which stimulates endogenous G protein–coupled receptors, resulting in decreased Ca^2+^ entry in TRPC1 KO mice indicating that TRPC1 is the functional store/receptor-operated Ca^2+^ entry (S/ROCE) channel in these cells (supplemental Fig. 1, *B* and *C*). Importantly, basal Ca^2+^ (no store depletion) was unaltered in adipocytes from WT or TRPC1 KO mice (supplemental Fig. 1, *D* and *E*). To establish the molecular identity of the Ca^2+^ entry channel, electrophysiological recordings were performed. Addition of thapsigargin induced an inward current which was nonselective and reversed between 0 and −5 mV (Fig. 1, *D–F*). Importantly, Ca^2+^ entry currents were significantly decreased in TRPC1 KO mice and the channel properties were similar to those previously observed with TRPC1 channels (23), suggesting that TRPC1 contributes to the endogenous store-mediated Ca^2+^ entry channel in adipocyte cells. Furthermore, we evaluated expression of other Ca^2+^ entry channels in adipocytes and found that along with TRPC1, TRPC5, STIM1, and Orai1 were also expressed in these cells (Fig. 1, *G* and *H*), although the properties of the store/receptor-operated Ca^2+^ entry in adipocytes was not inward rectifying as observed with Orai1-mediated I_CRAC_ channels (24, 25) suggesting that TRPC1 is the major Ca^2+^ channel in adipocytes. Together, these results suggest that TRPC1 is important for Ca^2+^ entry in adipocyte cells.

### Body fat mass is decreased in TRPC1 KO mice fed a HF diet and exercised

**Figure 2.**
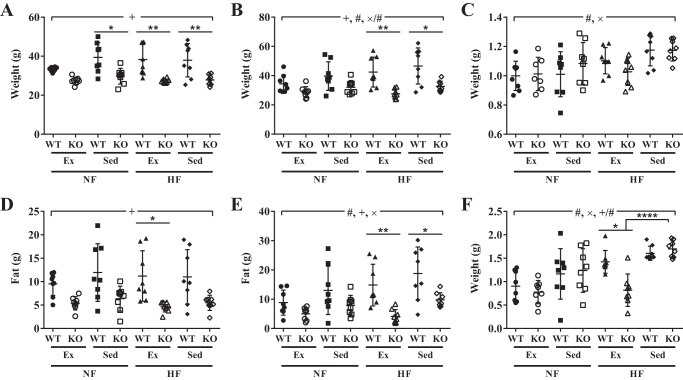
**TRPC1 KO mice fed a HF diet and exercised have decreased body fat mass.** *A* and *B*, body weight was measured at week 0 (*A*) and week 12 (*B*). *C*, body weight change was calculated by dividing the body weight at week 12 by the body weight at week 0. *D* and *E*, body fat mass was measured by EchoMRI at week 0 (*D*) and week 12 (*E*). *F*, body fat change was calculated by dividing the fat weight at week 12 by the fat weight at week 0. Data are presented as mean ± S.D., *n* = 6–8. Significant (*p* < 0.05) effects from 3-way ANOVA are indicated by + (mouse type), × (diet), and # (exercise). A significant interaction was further analyzed using post hoc Tukey to perform pairwise comparisons. *, *p* < 0.05; **, *p* < 0.01; ****, *p* < 0.0001.

TRPC1 KO mice had lower body weight (Fig. 2*A*) and body fat mass (Fig. 2*D*) at the start of the study and after 12 weeks of diet and exercise (Fig. 2, *B* and *E*) when compared with WT mice (*p* < 0.0001). However, when calculated as a -fold change, there was no change in body weight when comparing WT to TRPC1 KO mice (Fig. 2*C*), but body fat mass was significantly decreased (*p* < 0.05) in TRPC1 KO mice fed a HF diet and exercised compared with WT mice fed a HF diet and exercised (Fig. 2*F*). Furthermore, TRPC1 KO mice fed a HF diet and exercised had less body fat mass (*p* < 0.0001) than TRPC1 KO mice fed a HF diet and subjected to sedentary cage activity (Fig. 2*F*). Although food intake variation was influenced by the type of mouse (*p* < 0.01) and an exercise–diet interaction (*p* < 0.05), altered body composition was not a result of group differences in food consumption (*p* > 0.05) or exercise (*p* > 0.05) (supplemental Fig. 2, *A* and *B*). The data thus far show that TRPC1 is the major Ca^2+^ entry channel in adipocytes and that loss of TRPC1 decreases obesity risk in HF fed mice that exercise.

### TRPC1 KO mice fed a HF diet and exercised are protected from type II diabetes risk

**Figure 3.**
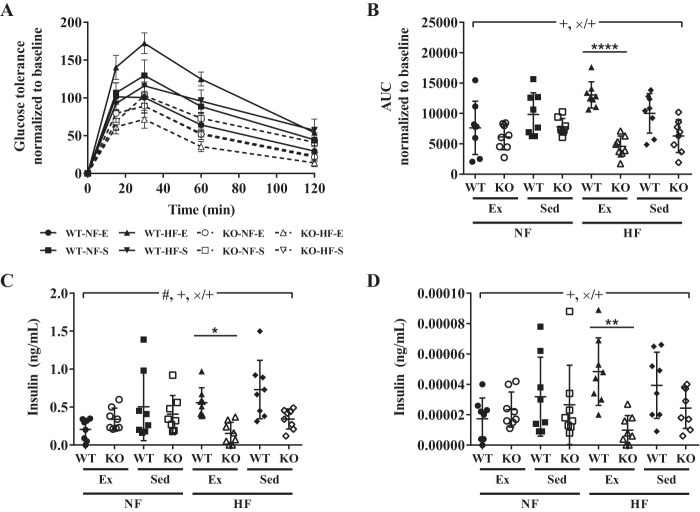
**TRPC1 KO mice fed a HF diet and exercised have reduced insulin resistance.** *A–D*, blood glucose (*A* and *B*), plasma insulin (*C*), and calculated homeostatic model assessment of insulin resistance (*D*) were measured from WT and TRPC1 KO mice fasted overnight after 12 weeks of diet and exercise. Data are presented as mean ± S.E. (*A*) or S.D. (*B–D*), *n* = 7–8. Significant (*p* < 0.05) effects from 3-way ANOVA are indicated by + (mouse type), × (diet), and # (exercise). A significant interaction was further analyzed using post hoc Tukey to perform pairwise comparisons. *, *p* < 0.05; **, *p* < 0.01; ****, *p* < 0.0001.

**Figure 4.**
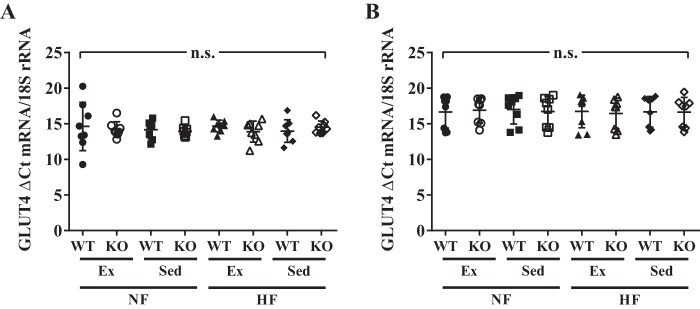
**GLUT4 expression is unaltered in subcutaneous adipose tissue and skeletal muscle.** *A* and *B*, GLUT4 expression in subcutaneous adipose tissue (*A*) and hind leg biceps femoris skeletal muscle (*B*) was measured from WT and TRPC1 KO mice following 12 weeks of diet and exercise. Data are presented as mean ± S.D., *n* = 7–8. No significant (*p* > 0.05) effects from 3-way ANOVA were identified. n.s., no significance.

The data provided above show an important role for TRPC1 in the onset of metabolic syndrome. Thus, glucose concentrations were next evaluated under these conditions. Maximum blood glucose concentrations occurred 15–30 min after intraperitoneal injection of glucose in all groups (Fig. 3*A*). However, blood glucose concentrations were decreased (*p* < 0.0001) in TRPC1 KO mice fed a HF diet and exercised when compared with WT mice fed a HF diet and exercised (Fig. 3*B*). Similarly, serum insulin concentrations were decreased (*p* < 0.05) in TRPC1 KO mice fed a HF diet and exercised compared with WT mice fed a HF diet and exercised (Fig. 3*C*). Using a homeostatic model assessment of insulin resistance (HOMA IR), we found that, once more, TRPC1 KO mice fed a HF diet and exercised were less insulin resistant (*p* < 0.01) than WT mice fed a HF diet and exercised (Fig. 3*D*) although this difference was not because of altered expression of GLUT4 in the subcutaneous adipose tissue (Fig. 4*A*) or skeletal muscle (Fig. 4*B*). These studies suggest that loss of TRPC1 decreases insulin resistance and risk of diabetes in HF fed mice that exercise thereby inhibiting metabolic syndrome.

### Adipocyte numbers are decreased in TRPC1 KO mice fed a HF diet and exercised

**Figure 5.**
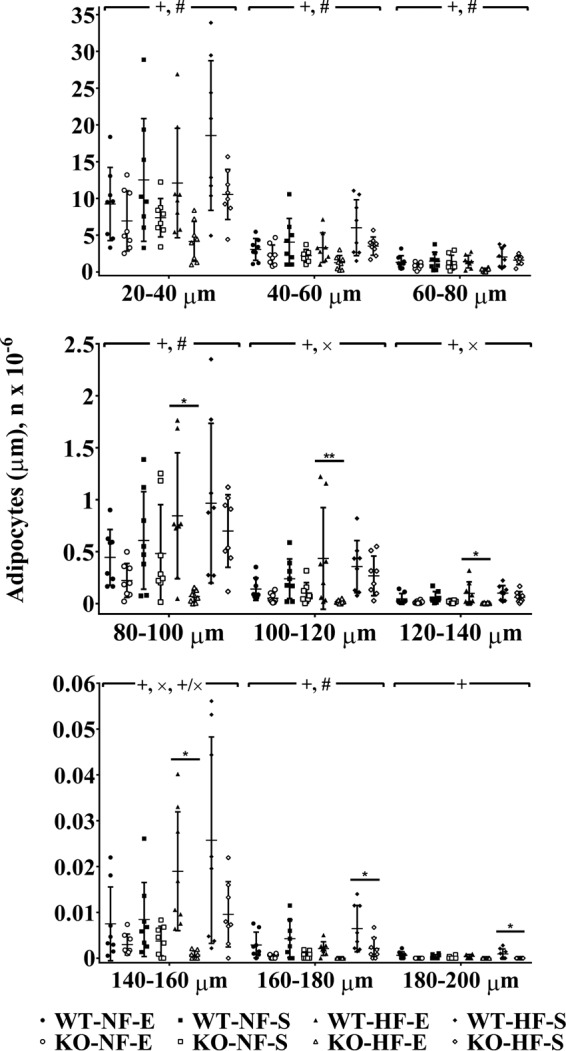
**TRPC1 KO mice fed a HF diet and exercised have fewer adipocytes.** Adipose tissue harvested from WT and TRPC1 KO mice following 12 weeks of diet and exercise was measured by a Multisizer. Data are presented as mean ± S.D., *n* = 6–8. Significant (*p* < 0.05) effects from 3-way ANOVA are indicated by + (mouse type), × (diet), and # (exercise). A significant interaction was further analyzed using post hoc Tukey to perform pairwise comparisons. *, *p* < 0.05; **, *p* < 0.01.

To establish if adipocyte number or size is varied under these conditions, we counted the number of adipocytes present in adipose tissue depots and determined the adipocyte size. In the subcutaneous and visceral adipose tissue depots, adipocytes with size ranges of 80–160 μm were decreased (*p* < 0.05) in TRPC1 KO mice fed a HF diet and exercised compared with WT mice fed a HF diet and exercised (Fig. 5). In addition, TRPC1 KO mice fed a HF diet and subjected to sedentary cage activity had decreased (*p* < 0.05) adipocytes from 160–200 μm when compared with WT mice fed a HF diet and subjected to sedentary cage activity (Fig. 5), suggesting that loss of TRPC1 decreases the number of larger adipocytes, which could result in the decreased fat mass observed in Fig. 2.

### Autophagy marker expression is decreased whereas apoptosis marker expression is increased in TRPC1 KO mice fed a HF diet and exercised

**Figure 6.**
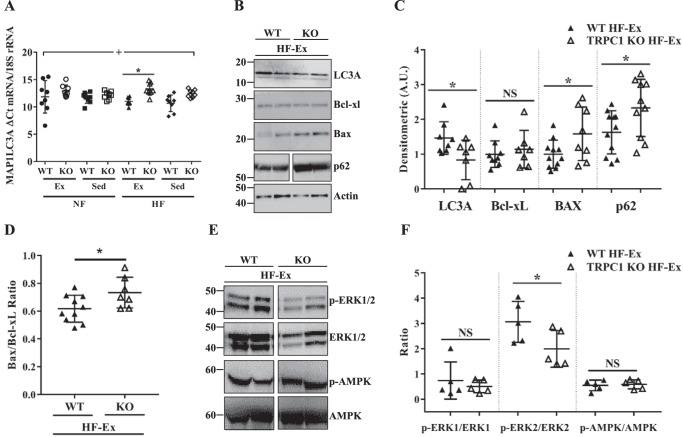
**Autophagy marker expression is decreased whereas apoptosis marker expression is increased in TRPC1 KO mice fed a HF diet and exercised.** *A*, MAP1LC3A mRNA expression was measured from subcutaneous adipose tissue taken from WT and TRPC1 KO mice following 12 weeks of diet and exercise. Data are presented as mean ± S.D., *n* = 6–8. Significant (*p* < 0.05) effects from 3-way ANOVA are indicated by + (mouse type). A significant interaction was further analyzed using post hoc Tukey to perform pairwise comparisons. *, *p* < 0.05. *B*, subcutaneous adipose tissue from WT and TRPC1 KO mice fed a HF diet and exercised was resolved and analyzed by Western blotting using antibodies labeled in the figure with β-actin as a loading control. Quantification of each protein is shown in *C*. Data are presented as mean ± S.D., *n* = 7–11. *, *p* < 0.05. *D*, ratio of Bax/Bcl-xl is presented as a -fold increase of TRPC1 KO over WT values. Data are presented as mean ± S.D., *n* = 7–10. *, *p* < 0.05. *E*, subcutaneous adipose tissue isolated from WT and TRPC1 KO mice fed a HF diet and exercised was resolved on SDS-PAGE gels and analyzed using different antibodies as labeled in the figure. *F*, quantification of the phosphorylated to non-phosphorylated form of each protein. Data are presented as mean ± S.D., *n* = 5. *, *p* < 0.05.

To determine whether reduced adipocyte numbers in adipose depots of TRPC1 KO mice fed a HF diet and exercised (Fig. 5) were because of apoptosis or reduced differentiation into adipocytes, we measured mRNA of key markers for adipogenesis (PPARγ (peroxisome proliferator–activated receptor γ)), beiging (FGF21 (fibroblast growth factor 21)), hypoxia (HIF1α (hypoxia-inducible factor 1-α)), and autophagy (MAP1LC3A (microtubule-associated proteins 1A/1B light chain 3A), BECN1 (beclin 1)). Although there was no altered mRNA expression of PPARγ, FGF21, HIF1α, or BECN1 in subcutaneous adipose tissue (supplemental Fig. 3, *A–D*), expression of the autophagy marker MAP1LC3A was decreased (as indicated by an increased threshold cycle (Ct) value) (*p* < 0.05) in TRPC1 KO mice fed a HF diet and exercised compared with WT mice fed a HF diet and exercised (Fig. 6*A*). To confirm that our mRNA expression was replicated on the protein level, we examined protein expression of autophagy (LC3A, p62) and apoptosis (Bax, Bcl-xl) regulating proteins in WT and KO mice fed a HF diet and exercised. LC3A expression was decreased along with an increase in p62 expression in samples from TRPC1 KO mice that were fed a HF diet and exercised when compared with WT mice fed a HF diet and exercised (Fig. 6, *B* and *C*). Similarly, increased expression of Bax and an increased Bax to Bcl-xl ratio was observed in the TRPC1 KO mice fed a HF diet and exercised compared with WT mice fed a HF diet and exercised (Fig. 6, *B–D*). Together, these results suggest that loss of TRPC1 decreases autophagy, a survival mechanism, and increases apoptosis, which could promote loss of larger adipocytes. In addition, loss of TRPC1 significantly decreased phosphorylation of ERK2, whereas no change in the phosphorylation of ERK1 and AMPK was observed (Fig. 6, *E* and *F*). These data further indicate that loss of TRPC1 inhibits ERK2 phosphorylation, which has been shown to interact with ATG proteins and thus could modulate autophagy in these cells.

## Discussion

This study is the first to show that TRPC1 KO mice that exercise are protected from HF diet-induced obesity and type II diabetes risk because of decreased adipose tissue mass and adipocyte number as a result of reduced autophagy and increased apoptosis. Thus, in combination, exercise, HF diet, and loss of TRPC1 reduce adiposity through a yet undefined mechanism. Given that TRPC1 is involved in Ca^2+^ entry following depletion of internal Ca^2+^ stores in the ER, TRPC1 KO results in decreased Ca^2+^ entry in a variety of cell types including adipocytes (3), skeletal muscle (4), neuronal (26), intestinal epithelial cells (27), and salivary glands (23, 28). Thus, based on our data, it is probable that reduced Ca^2+^ entry due to TRPC1 KO is influenced further by HF diet and exercise, suggestive of a relationship between Ca^2+^ entry, diet, and exercise. Although expression of Orai1 and STIM1 was observed in adipocytes, the properties of the endogenous channel were similar to those observed with TRPC1-mediated I_SOC_ (23, 28) and not as observed with I_CRAC_ channels (24, 25). Furthermore, TRPC1 KO mice showed exercise-mediated inhibition of adiposity and decreased insulin resistance in the absence of TRPC1 suggesting that TRPC1 might be the dominant Ca^2+^ channel in these cells. However, additional studies will be needed to determine the role of Orai1 channels in exercise-mediated regulation of metabolic syndrome.

The present study demonstrated that fat mass was reduced in TRPC1 KO mice compared with WT mice following 12 weeks of HF diet and exercise. Similarly, previous studies have shown that TRPV4 KO mice (another Ca^2+^ entry channel) are also protected from obesity and metabolic dysfunction with exposure to HF diet (18) suggesting that Ca^2+^ channels negatively regulate obesity. This is in contrast to the expectations that HF-fed mice develop obesity and glucose intolerance (29) because TRPC1 KO mice fed a HF-diet and exercised were less insulin resistant than their WT counterparts, indicative of protection from type II diabetes risk, yet GLUT4 expression was unaltered in hind leg biceps femoris skeletal muscle or subcutaneous adipose tissue. Furthermore, the number and size of subcutaneous and visceral adipocytes are decreased in TRPC1 KO mice compared with WT mice when fed a HF diet and exercised. Because TRPC1 plays a key role in cell survival and apoptosis (30–32), it was hypothesized that TRPC1 KO mice would alter expression patterns of key markers for adipogenesis, apoptosis, or autophagy in subcutaneous adipose tissue. TRPC1 KO mice fed a HF diet and exercised had decreased expression of the autophagy marker MAP1LC3A along with an increase in apoptosis markers (particularly the ratio of Bax/Bcl-xl), which is in agreement with our previous findings that silencing of TRPC1 decreased autophagy and increased cell death (33). Loss of TRPC1 also decreased phosphorylation of ERK2, which is consistent with previous studies where activation of Ca^2+^ channels in adipocytes increased ERK2 phosphorylation (18). In addition, loss of TRPC1 decreased the number of larger adipocytes. These findings suggest that elimination of TRPC1-mediated Ca^2+^ entry in TRPC1 KO mice promotes suppression of autophagy in HF diet-fed and exercised mice resulting in increased adipocyte cell death. These results are consistent with previous studies where patients with metabolic syndrome also have higher serum Ca^2+^ levels (34, 35), which could be because of the loss of TRPC1 or other Ca^2+^ channels that mediate Ca^2+^ entry in adipocyte cells, thereby increasing serum Ca^2+^ levels.

Interestingly, in skeletal muscle, even though contraction does not depend on extracellular Ca^2+^ (36), Ca^2+^ entry through TRPC1 is essential for maintaining force during sustained repeated contractions as TRPC1 KO mice experience muscle fatigue during endurance exercise though spontaneous wheel running activity is unchanged (4). Our data are in agreement as we showed no alteration in voluntary exercise. However, a reduction in endurance exercise might be expected because loss of TRPC1 could impact mitochondrial respiration by altering Ca^2+^ homeostasis, because of an increase in total mitochondrial protein stimulated by exercise training (37, 38), and Ca^2+^ is needed for proper functioning of mitochondria (39). In addition, ER stress resulting from reduced Ca^2+^ entry could increase translocation of apoptotic factors into mitochondria thus permeabilizing the membrane, causing release of cytochrome *c* and activation of caspases, leading to mitochondria-mediated cell death (30, 40–42). These findings demonstrate that loss of TRPC1 disrupts Ca^2+^ homeostasis potentially resulting in mitochondria-mediated cell death of adipocytes. Although a previous study has shown that knock down of TRPC1 only attenuated nonstimulated Ca^2+^ influx in breast cancer cells (43), our results using adipocytes did not show any decrease in basal Ca^2+^ entry. These results suggest that although in breast cancer cells other Ca^2+^ influx channels (Orai1) might be more important for SOCE, TRPC1 is essential for adipocyte function, especially in blocking the effects of exercise in HF diet-induced obesity. The mechanism by which TRPC1 KO mice fed a HF diet and exercised are protected from obesity and type II diabetes risk needs further investigation. However, our study and another published study (18) indicate that loss of Ca^2+^ might be the main factor that inhibits the formation of metabolic syndrome.

## Experimental procedures

### Study design and animals

Four-month-old male B6129SF2/J (WT) or TRPC1 knock-out (KO) mice (The Jackson Laboratory, Bar Harbor, ME) were fed diets containing either 16% (normal-fat (NF)) or 45% fat (high-fat (HF)) for 12 weeks and subjected to voluntary wheel running exercise (exercise (E)) or sedentary cage activity (sedentary (S)). Experimental groups were labeled according to diet and exercise conditions yielding eight groups: WT-NF-E, WT-NF-S, WT-HF-E, WT-HF-S, KO-NF-E, KO-NF-S, KO-HF-E, and KO-HF-S. Food intake, body weight, and body composition were measured biweekly on alternating weeks during the experimental feeding period. After 12 weeks, mice were injected with xylazine (Akorn Inc., Decatur, IL) and ketamine (Zoetis Inc., Kalamazoo, MI) and then killed by exsanguination according to the animal use and care protocol approved by the USDA Agricultural Research Service Animal Care and Use Committee.

### Calcium measurements and electrophysiology

Primary adipocyte cells were incubated with 2 μm fura-2 (Molecular Probes) for 45 min and then washed twice with Ca^2+^-free SES buffer as described in Liu *et al.*(27). For patch clamp experiments, coverslips with cells were transferred to the recording chamber and perfused with an external Ringer's solution of the following composition (mm): NaCl, 145; KCl, 5; MgCl_2_, 1; CaCl_2_, 1; Hepes, 10; Glucose, 10; pH 7.4 (NaOH). The patch pipette had resistances between 3 and 5 megaohms after filling with the standard intracellular solution that contained the following (mm): cesium methane sulfonate, 145; NaCl, 8; MgCl_2_, 10; Hepes, 10; EGTA, 10; pH 7.2 (CsOH). The maximum peak currents were calculated at a holding potential of −80 mV. The current-voltage (I/V) curves were made using a ramp protocol where current density was evaluated at various membrane potentials and plotted. For the tabulation of statistics, peak currents were used.

### EchoMRI measurements of body composition

Whole body composition, including fat mass and lean mass, was determined biweekly during the 12-week period without sedation using nuclear magnetic resonance technology with the EchoMRI700™ instrument (Echo Medical Systems, Houston, TX).

### Glucose tolerance test

At the end of 12 weeks of feeding, mice were fasted overnight and then injected with 2 g/kg body weight of 20% d-glucose (Sigma-Aldrich) intraperitoneally. Approximately 5 μl of tail blood was used to measure the blood glucose concentrations as described previously (14, 44) using the Accu-Chek Aviva glucometer at baseline and then 15, 30, 60, and 120 min post glucose injection.

### Measurement of plasma insulin

Mice were fasted overnight and then plasma was obtained to analyze insulin concentrations (Insulin ELISA kit: EXRMI-13K, EMD Millipore, St. Charles, MO) as previously described (12) using the Bio-Rad Luminex system according to manufacturer's protocols.

### Stromal vascular fraction (SVF) and primary adipocyte isolation

Subcutaneous and visceral adipose tissue were weighed and digested as described previously (12–14). Briefly, following digestion with collagenase type I (Gibco Thermo Fisher Scientific) at 37 °C for 1 h, adipose tissue cells were filtered using 100-μm nylon cell strainers (Corning Life Sciences) followed by centrifugation (1000 rpm, 10 min, 4 °C) to separate floating primary adipocytes (supernatant) from adipose SVF (cell pellet). The SVF cell pellet was treated with RBC lysis buffer (Sigma Aldrich) then quenched with DMEM + 10% FBS and the supernatant was washed and resuspended in 0.9% NaCl for adipose cell size and number determination using a Beckman Coulter Multisizer 4 with a 400-μm aperture. The instrument was set to count 6000 particles and the cell suspension was diluted so that coincident counting was <10%. After collection of pulse sizes, the data were expressed as cell numbers per particle diameter.

### PCR analysis

Total RNA was extracted using the RNeasy Lipid Tissue Mini kit and Qiacube (Qiagen, Valencia, CA) from flash-frozen hind leg biceps femoris skeletal muscle or subcutaneous adipose tissue. cDNA was synthesized using the Quantitect Reverse Transcriptase kit (Qiagen, Valencia, CA) and then used to measure expression of GLUT4, HIF1α, FGF21, PPARγ, MAP1LC3A, and BECN1 by qPCR (ABI Prism 7500 PCR System, Applied Biosystems, Foster City, CA). FastStart Universal Probe Master (Rox) mix assay reagents were purchased from Roche. Primers were purchased from Integrated DNA Technology (IDT) (Coralville, IA). The endogenous control (18S rRNA) was purchased from Applied Biosystems. RT-PCR analysis for TRPC1 transcripts was done with primers from the eighth and ninth exons (forward, 5′-GCAACCTTTGCCCTCAAAGTG and reverse, 5′-GGAGGAACATTCCCAGAAATTTCC) after the EcoRI site (Eurofins MWG Operon, Huntsville, AL).

### Protein extraction and immunoblotting

Protein was extracted from subcutaneous adipose tissue of WT and KO mice fed a HF diet and exercised, as described previously (13). 40 μg of proteins were resolved on NuPAGE Novex 4–12% Bis-Tris gels, transferred to nitrocellulose membranes, and probed with respective antibodies (all from Cell Signaling Technology). Respective peroxidase-conjugated secondary antibodies were used to label the proteins, which were then detected by an enhanced chemiluminescence detection kit (SuperSignal West Pico, Pierce). Densitometric analysis was performed using ImageJ (National Institutes of Health).

### Statistical analysis

Data are reported as mean ± S.D. or mean ± S.E. The effects of TRPC1 KO, diet, or exercise were assessed by three-way analysis of variance (ANOVA) using GraphPad Prism 7. Statistical significance is denoted as + (mouse type), × (diet), and # (exercise) for main ANOVA interactions. When an interaction was significant (*p* < 0.05), Tukey contrasts were used to perform pairwise comparisons, which are reported as follows: *, *p* < 0.05; **, *p* < 0.01; ***, *p* < 0.001; ****, *p* < 0.0001.

## Author contributions

B. B. S., J. N. R., and K. J. C.-L. designed the studies that were performed by D. K., A. S., Y. S., and P. S. The paper was written by D. K., A. S., B. B. S., J. N. R., and K. J. C.-L. All authors reviewed the results and approved the final manuscript.

## Supplementary Material

Supplemental Data

## Footnotes

4 The abbreviations used are:

TRPC1: transient receptor potential canonical channel-1
TRPV: vanilloid receptor-related TRP channels
HF: high fat
ER: endoplasmic reticulum
NF: normal-fat.
