# Supplementary material for: The TRPC1 Ca2+-permeable channel inhibits exercise-induced protection against high-fat diet-induced obesity and type II diabetes
Source: J Biol Chem. 2017 Oct 26;292(50):20799–807. doi: 10.1074/jbc.M117.809954 (PMC5733613; doi:10.1074/jbc.M117.809954)
Supplement: Supplemental Data [file supp_292_50_20799__index.html]

The TRPC1 Ca2+-permeable channel inhibits exercise-induced protection against high-fat diet-induced obesity and type II diabetes — The TRPC1 Ca2+-permeable channel inhibits exercise-induced protection against high-fat diet-induced obesity and type II diabetes — TRPC1 deficiency protects against metabolic syndrome — Supplemental Data 

# The TRPC1 Ca2+-permeable channel inhibits exercise-induced protection against high-fat diet-induced obesity and type II diabetes

## Supplemental Data

- Supplemental (.docx, 149 KB) - Supplemental
