# Supplementary material for: The TRPC1 Ca2+-permeable channel inhibits exercise-induced protection against high-fat diet-induced obesity and type II diabetes
Source: J Biol Chem. 2017 Oct 26;292(50):20799–807. doi: 10.1074/jbc.M117.809954 (PMC5733613; doi:10.1074/jbc.M117.809954)
Supplement: Supplemental Data [file 10.1074_M117.809954_jbc.M117.809954-1.docx]

**SUPPLEMENTAL INFORMATION**

**FIGURE 1.** (A) Peak ratio from Figure 1B (second peak representing Ca^2+^ entry compared to first peak representing store depletion upon addition of thapsigargin) is shown in 30-40 adipocytes obtained from WT and TRPC1 KO mice. *** indicates significance (*p* < 0.001). Analog plots of the fluorescence ratio (340/380) that represent Ca^2+^ entry upon addition of angiotensin in a Ca^2+^ containing media from an average of 30-50 cells isolated from WT or TRPC1 KO mice are shown in B. Quantification (mean ± S.D.) of 340/380 ratio under these conditions is shown in C. *** indicates significance (*p* < 0.001). Analog plots of the fluorescence ratio (340/380) that represent basal Ca^2+^ entry (without store-depletion) from an average of 40-50 cells in each condition are shown in D. (E) Quantification (mean ± S.D.) of 340/380 ratio.

**FIGURE 2. Daily food intake and exercise is unaltered.** Food consumption and exercise were measured biweekly from WT and TRPC1 KO mice over the course of 12 weeks. Data are presented as means ± S.D., n = 7-8. Significant (*p* < 0.05) effects from 3-way ANOVA are indicated by + (mouse type), × (diet), and # (exercise). A significant interaction was further analyzed using post hoc Tukey to perform pairwise comparisons, but there was no significance detected between groups. n.s., no significance.

**FIGURE 3. Expression of markers for adipogenesis, beiging, and hypoxia were unaltered in subcutaneous adipose tissue.** mRNA expression of PPARγ (adipogenesis) (A), FGF21 (beiging) (B), HIFIα (hypoxia) (C), and BECN1 (autophagy) (D) was measured from subcutaneous adipose tissue taken from WT and TRPC1 KO mice following 12 weeks of diet and exercise. Data are presented as means ± S.D., n = 6-8. No significant (*p* > 0.05) effects from 3-way ANOVA were identified. n.s., no significance.
